# Influence of under pressure dissolved oxygen on trichloroethylene degradation by the H_2_O_2_/TiO_2_ process

**DOI:** 10.1186/2052-336X-11-38

**Published:** 2013-12-20

**Authors:** Mohammad Hoseini, Ramin Nabizadeh, Shahrokh Nazmara, Gholam Hossein Safari

**Affiliations:** 1 Department of Environmental Health Engineering, School of Public Health, Tehran University of Medical Sciences, Poursina St, Tehran, Iran; 2 Center for Air Pollution Research, Institute for Environmental Research, Tehran University of Medical Sciences, Krgar St, Tehran, Iran

## Abstract

**Background:**

The widespread use of trichloroethylene (TCE) and its frequent release into the environment has caused many environmental and health problems. In this study the degradation of TCE at different micromolar concentrations was investigated in a stainless steel reactor with various concentrations of H_2_O_2_ and TiO_2_ at different oxygen pressures and three different pHs.

**Methods:**

To examine the synergistic effect of under pressure oxygen on TCE degradation, the concentrations of H_2_O_2_ and TiO_2_ as well as pH were first optimized, and then the experiments were performed under optimal conditions. Gas chromatography with a flame ionization detector (FID) was used to measure TCE concentrations.

**Results:**

Results showed that the percentage of TCE degradation without pressurized oxygen was low and it increased with increasing pressure of oxygen at all initial concentrations of TCE. The degradation percentages without oxygen pressure were 48.27%, 51.22%, 58.13% and 64.33% for TCE concentrations of 3000, 1500, 300 and 150 μg/L respectively. At an oxygen pressure of 2.5 atmospheres (atm) the percent degradation of TCE reached 84.85%, 89.14%, 93.13% and 94.99% respectively for the aforementioned TCE concentrations.

**Conclusions:**

The results of this study show that the application of dissolved oxygen under pressure increases the efficiency of the H_2_O_2_/TiO_2_ process on the degradation of TCE and can be used along with other oxidants as an effective method for the removal of this compound from aqueous solutions.

## Background

TCE is described by the US Environmental Protection Agency (EPA) as a halogenated aliphatic organic compound which has been widely used as an industrial solvent in applications including dry cleaning, paint stripping and chemical, pharmaceutical, and plastic manufacturing. It is used mainly as a degreasing agent because of its unique properties and solvent effects [1,2].

It is estimated that the production of TCE in 1990 was approximately 131 kilotons in Western Europe, 79 kilotons in the United States of America and 57 kilotons in Japan and its annual consumption in these areas was estimated as 65-103% of production levels [3]. Due to its widespread use and physical characteristics, TCE may enter into water supplies and groundwater. Drinking water supplies that use groundwater sources contaminated with TCE may contain this compound [1]. According to reports from the Agency for Toxic Substances and Disease Registry (ATSDR) TCE is the most frequently reported organic contaminant in groundwater in the US. The TCE-contaminated drinking water supply sources in the US are estimated at between 9-34% of total water supply sources [1,4].

It has been reported that exposure to TCE has many adverse effects on human health. Most of the reported effects have been associated with the effects of TCE on the central nervous system, with reported symptoms of fatigue, sleepiness, headache, confusion, and blurred vision [1,4]. Other effects on the liver, kidneys, gastrointestinal tract, and skin have also been reported [5]. According to US.EPA reports, exposure to TCE is associated with cancer of the kidneys and other organs [1]. It was also reported that TCE can cause loss of hearing in laboratory animals [4]. This compound induces cancer in mice and rats and it is considered as a probable carcinogenic chemical (Group B_2_) to humans [6].

Conventional water and wastewater treatment processes such as coagulation, sedimentation, precipitative softening, filtration and chlorination have failed to reduce concentrations of TCE to nonhazardous levels [7]. Treatment technologies such as adsorption by activated carbon and air stripping are effective in removing TCE from contaminated waters. Nakano et al. however, believe that transformation of TCE from one phase to another phase would be the most disadvantageous of these processes [8].

During the past several years numerous studies have been performed to investigate various techniques and technologies for the removal of this pollutant from contaminated water. Amongst different kinds of degradation and removal methods, advanced oxidation processes (AOPs) provide an effective means of rapidly treating this pollutant with efficient process control [7,9]. There are some reports regarding TCE degradation by various advanced oxidation processes such as H_2_O_2_ /iron (II) (Fenton’s reaction) [9], ultrasound/H_2_O_2_[10], gamma-rays/O_3_ and H_2_O_2_ /gamma-rays [11]. Cross et al. investigated the effects of the direct addition of dissolved oxygen on the combination of copper ions and ascorbic acid in an oxidation process known as a modified Fenton reagent. They reported that dissolved oxygen has a considerable synergistic effect on this system’s efficiency [12]. The main objective of this work was to investigate the synergetic effect of under pressure dissolved oxygen (UPDO) on TCE degradation by H_2_O_2_ and TiO_2_ nano-particles. The effects of different operating conditions such as TiO_2_ and H_2_O_2_ concentration, pH, and oxygen pressure on TCE removal efficiency were explored.

## Materials and methods

### General procedures

This experimental study was conducted in bench scale and batch system on synthetic solutions containing different concentrations of TCE. Aqueous solutions with different initial concentrations of TCE (150, 300, 1500, and 3000 μg/L) were prepared by dissolving TCE (Merck Co., Germany- Cat. No. 100958) in distilled and deionized (DD) water. The selection of these concentrations was based on TCE levels found in underground water in Tehran which ranged from 97.7 to 1345.7 μg/L [13]. TiO_2_ particles (P25, Degussa AG, Germany) with a primary particle diameter of 21 nm, specific surface area of 50 ± 15 m^2^/ g, and a crystal distribution of 80% anatase and 20% rutile were used as the catalyst for the experiments and dosed in the range of 25–200 mg/L. Four different concentrations (10, 25, 50, and 100 mg/L) of H_2_O_2_ were made by dissolving 30% H_2_O_2_ (Merck Co., Germany- Cat. No. 108597) in samples and concentrations were validated by iodometric back titration with 0.1 N sodium thiosulfate [9]. For adjustments of pH, NaOH and HCl supplied from Merck Co. were used and the pH values were measured using a pH meter (Metrohm E520). The prepared TCE solutions along with the given concentrations of TiO_2_ and H_2_O_2_ were exposed to different oxygen pressures (1, 1.5, 2, and 2.5 atm) at different reaction times of 15, 30, 45, 60, 90, and 120 minutes. For each run, the experiments were conducted three times and the averages were reported.

### Pilot set-up

**Figure 1:**
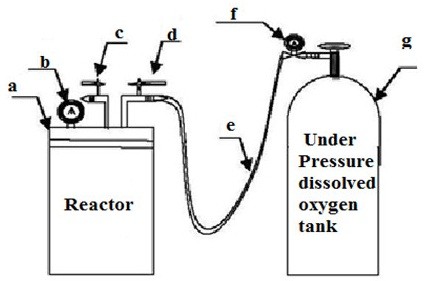
Schematic representation of the pilot set-up: a- Screw cap, b- Pressure gauge, c- Ball valve for gas pressure regulation, d- Ball valve for oxygen gas entrance, e- Connector hose, f- Pressure reducing Valve, g- Oxygen cylinder.

All experiments were performed in a 6 L stainless steel reactor which was connected by a plastic pipe to a cylinder containing 99.9% pure oxygen (Figure 1). After the addition of 2 L of prepared solution to the reactor and the application of appropriate doses of H_2_O_2_ and/or TiO_2_ nano-particles, the reactor was completely sealed to minimize any losses of TCE due to volatilization. However, in order to determine further losses, blank samples (i.e., unexposed control samples) were analyzed routinely. Then, the real concentration of each sample was calculated by subtracting the blank values from the results of the exposed samples. The oxygen gas entrance valve (marked *d* in Figure 1) was then opened until the pressure gauge displayed the desired pressure. Adjustment of the pressure was done manually by opening and closing the gas pressure regulating valve (*c* in Figure 1) and the gas entrance valve. To provide continuous agitation, the reactor was placed on a shaker.

### Analysis

The TCE concentrations were measured using a Varian CP–3800 (Australia) gas chromatograph (GC), equipped with an FID and a 30 m × 0.32 mm CP–Sil & CB capillary column with a film thickness of 0.25 μm. The initial oven temperature of 35°C was increased at a rate of 16°C/min to a final temperature of 100°C. The injector temperature was 150°C. The inlet was operated in 20% split mode. Helium (99.999%) was used as the carrier gas at a rate of 1 mL/min.

## Results and discussion

### Effect of H_2_O_2_ concentration

**Figure 2:**
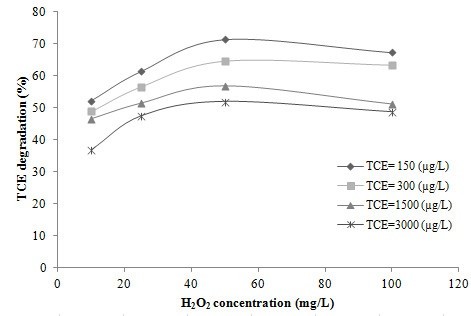
**TCE removal at different H**_**2**_**O**_**2 **_**concentrations; TiO**_**2 **_**concentration = 50 mg/L, oxygen pressure = 1 atm, pH = neutral and time = 45 min.**

Aqueous solutions with different initial concentrations of TCE and different concentrations of H_2_O_2_ (10, 25, 50 and 100 mg/L) at neutral pH, and a TiO_2_ concentration of 50 mg/L were exposed to UPDO (1 atm) for 45 min. The removal rate of TCE versus H_2_O_2_ concentration is shown in Figure 2. As an overall trend it is clear that the percentage of degradation for all TCE concentrations increased quite rapidly with the increase in H_2_O_2_ concentration until 50 mg/L and that at concentrations higher than this the TCE removal rate decreased. Apparently, the quenching of OH^•^ radicals according to the below quenching reactions causes the reduction in TCE degradation at H_2_O_2_ concentrations above 50 mg/L. Quenching reactions may include [9]:

(1) $$OH^{•}+H_{2}\;o_{2}\;\to \;HO_{2}{}^{•}+H_{2}O$$

(2) $$OH^{•}+HO_{2}{}^{{}_{{}_{{}^{•}}}}\;\to \;O_{2}+H_{2}O$$

It appears that in the advanced oxidation processes based on hydrogen peroxide, the effectiveness of H_2_O_2_ on the degradation of TCE depends on the type of process and the other available oxidants that may be used as synergists; for example Jung et al. found that in the radiation treatment of TCE and tetrachloroethylene (PCE) by gamma-rays, the presence of H_2_O_2_ did not affect the decomposition process [11]. However, it is reported that degradation of TCE by sonication in the presence of H_2_O_2_ is dependent upon the H_2_O_2_ concentration. The best TCE degradation rate was achieved at an H_2_O_2_ concentration of 10 mg/L and increasing the H_2_O_2_ concentration from 10 to 50 mg/L did not affect the TCE destruction [10]. Therefore, there is a maximum level of H_2_O_2_ beyond which any improvement in the degradation rate is decreased; and in our experiments the maximum level was 50 mg/L.

### Effect of TiO_2_ concentration

**Figure 3:**
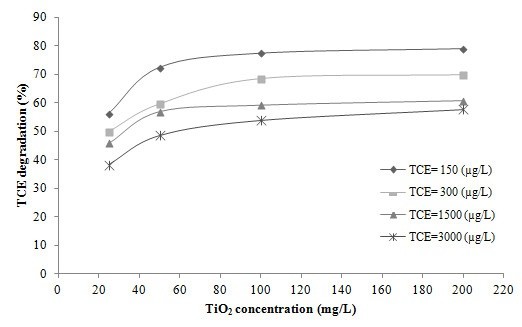
**Degradation of TCE at different TiO**_**2**_** concentrations.** H_2_O_2_ concentration = 50 mg/L, oxygen pressure = 1 atm, pH = neutral and time = 45 min.

Figure 3 shows the removal of TCE at different concentrations of TiO_2_ (25, 50, 100 and 200 mg/L) at neutral pH, 50 mg/L H_2_O_2,_ 1 atm of oxygen and a reaction time of 45 min. As shown in this figure, the TCE removal increased with an increase in TiO_2_ concentration and reached a plateau at a TiO_2_ concentration of 100 mg/L, after which it remained approximately stable. This result is similar to the results reported by other researchers who studied the effect of TiO_2_ nano-particles on the degradation of other organic pollutants in various AOPs [10,14,15]. Yamazaki et al. compared the effectiveness of Degussa P-25 TiO_2_ nano-particles with the commercially available PC-101 and PC-102 powders in addition to SG powders, which are prepared by the sol–gel method of photocatalytic degradation of TCE in water. They found that the degradation rate of TCE using P-25 was about three-fold higher than when the same experiments were performed with the other three aforementioned nano-particle powders. Differences in the degradation rates were mainly due to different specific surface area, crystal structure, and specific volume of the various TiO_2_ powders [2]. Some studies have determined that in the AOPs which use TiO_2_ nano-particles as catalysts for the removal of organic pollutants, when the concentration of nano-particles exceeds a certain value, the activated TiO_2_ may deactivate through collisions with ground-state catalysts. Additionally, agglomeration and sedimentation of TiO_2_ occurs in the presence of high concentrations of catalyst [16-18]. Therefore, lack of any increase in TCE removal efficiency at TiO_2_ concentrations higher than 100 mg/L in our work can be attributed to deactivation, agglomeration and sedimentation of TiO_2_ nano-particles. Avoiding these problems is essential to the optimization of TiO_2_ concentration.

### Effect of pH

**Figure 4:**
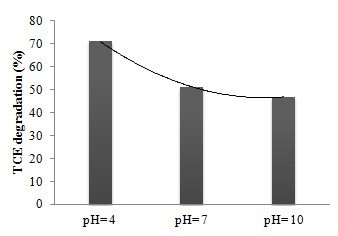
**Effect of pH on TCE degradation.** TCE = 1500 μg/L, TiO_2_ = 100 mg/L, H_2_O_2_ = 50 mg/L, oxygen pressure = 1 atm, and time = 45 min.

In AOPs, pH can influence the pollutant degradation rate. As shown in Figure 4, there was a significant difference in TCE degradation at various pHs; and the degradation was significantly higher in acidic pH. Similar results were obtained by Teel et al. who found that TCE degradation in goethite-catalyzed reactions increased significantly with decreasing pH. They also reported that the degradation rate of pentachlorophenol in Fenton’s reactions decreased significantly with increasing pH [9]. Although acidic conditions appear to enhance catalysis by TiO_2_ in the reactor, the underlying mechanism for this effect is unknown. In addition, under some conditions H_2_O_2_ based reactions are highly redox sensitive, and OH^•^ formation due to H_2_O_2_ decomposition increases significantly under the reducing conditions of low pH.

### Effect of reaction time and initial TCE concentration

To investigate the effect of reaction time and initial TCE concentration on the TCE removal rate, given concentrations of TCE were exposed to four different oxygen pressures during six reaction times with the optimized values of H_2_O_2_ concentration (50 mg/L), TiO_2_ loading (100 mg/L) and pH value (4). Table 1 shows the fitted equations and R^2^ value relative to percent TCE degradation versus reaction time for different oxygen pressures. As shown in this table the best fit trend lines for percent degradation versus reaction time were polynomial. The results showed that the degradation percent increased with increasing reaction time from 15 to 90 min and then stayed approximately constant. Decreasing the initial concentration of TCE caused an increase in removal rate. For example, at an oxygen pressure of 1 atm, initial TCE concentration of 150 μg/L, and contact time of 90 minutes, 87.21% (± 6.82%) TCE degradation was achieved; whereas for concentrations of 300, 1500 and 3000 μg/L, the average (± S.D.) of TCE removal efficiency were 83.13% (± 7.23%), 75.28% (± 5.65%) and 71.46% (± 5.65%) respectively. This trend was also observed at other oxygen pressures. This may be explained by the fact that under the same conditions, the OH° radical densities were equal in all solutions; therefore, the reaction of TCE with OH° radicals becomes more likely at lower TCE concentrations, resulting in an overall increase in TCE degradation by OH° radicals [10]. This result was in agreement with results reported by other researchers who studied TCE degradation by various AOPs. For example, Huang et al. studied the potential of gamma radiation technology for TCE degradation and found that with increasing initial concentration of TCE, increased time and gamma-ray dosage was required for the degradation process [19].

**Table 1:** **Fitted equations and R**^**2 **^**related to percent TCE degradation versus reaction time for different oxygen pressures under optimized conditions**

**Oxygen (O**_**2**_**) pressure (atm)**	**TCE (μg/L)**	**Fitted equation**	**R**^**2**^
1	150	y = 4E-05x^3^ - 0.014x^2^ + 1.641x + 23.40	0.999
	300	y = 4E-05x^3^ - 0.014x^2^ + 1.735x + 16.82	0.995
	1500	y = 5E-05x^3^ - 0.015x^2^ + 1.680x + 14.22	0.997
	3000	y = 5E-05x^3^ - 0.015x^2^ + 1.675x + 10.45	0.995
1.5	150	y = 6E-05x^3^ - 0.018x^2^ + 1.894x + 25.84	0.998
	300	y = 5E-05x^3^ - 0.014x^2^ + 1.630x + 24.96	0.996
	1500	y = 2E-05x^3^ - 0.009x^2^ + 1.310x + 23.36	0.999
	3000	y = 2E-05x^3^ - 0.010x^2^ + 1.455x + 13.85	0.999
2	150	y = 3E-05x^3^ - 0.010x^2^ + 1.307x + 41.45	0.996
	300	y = 3E-05x^3^ - 0.011x^2^ + 1.476x + 32.36	0.996
	1500	y = 4E-05x^3^ - 0.014x^2^ + 1.651x + 24.65	0.995
	3000	y = 4E-05x^3^ - 0.012x^2^ + 1.442x + 24.79	0.999
2.5	150	y = 3E-05x^3^ - 0.010x^2^ + 1.307x + 41.45	0.996
	300	y = 3E-05x^3^ - 0.011x^2^ + 1.476x + 32.36	0.996
	1500	y = 4E-05x^3^ - 0.014x^2^ + 1.651x + 24.65	0.995
	3000	y = 4E-05x^3^ - 0.012x^2^ + 1.442x + 24.79	0.999

### Effect of under pressure oxygen

**Figure 5:**
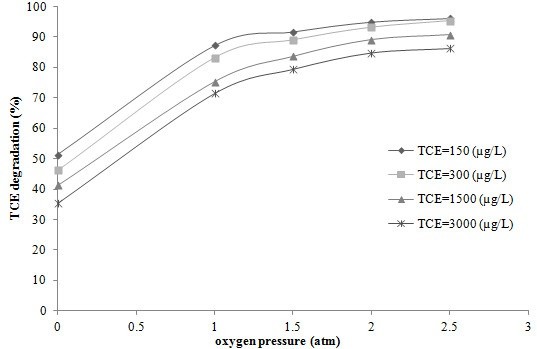
**Degradation of TCE in various oxygen pressures (H**_**2**_**O**_**2**_ **= 50 mg/L, TiO**_**2**_ **= 100 mg/L, pH = 4 and reaction time 90 min).**

**Figure 6:**
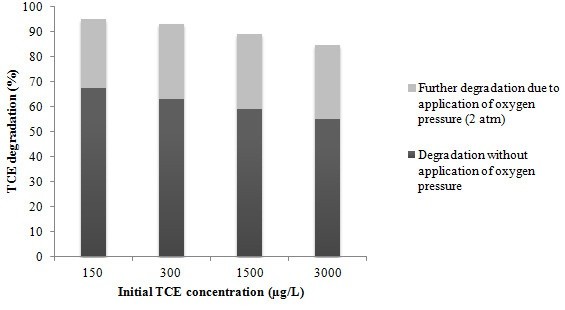
Additional removal of TCE due to the application of an oxygen pressure of 2 atm under optimum conditions.

Figure 5 shows the TCE removal percentage versus oxygen pressure for various initial concentrations of TCE following a 90 min reaction time with optimized values of H_2_O_2_, TiO_2_ and pH; and Figure 6 shows the additional removal of TCE due to the application of 2 atm of oxygen under optimum conditions. According to Figure 5, TCE degradation increased rapidly with increasing oxygen pressure. It can be clearly seen that without applying oxygen, the overall degradation of TCE is low. Without oxygen pressure the percentages of TCE degradation were 48.27%, 51.22%, 58.13% and 64.33% for TCE concentrations of 3000, 1500, 300 and 150 μg/L, respectively. These degradation percentages increased markedly to 71.46%, 75.28%, 83.13% and 87.21% at 1 atm of oxygen. These values gradually continued to increase up to an oxygen pressure of 2 atm. At this pressure the degradation rates were 84.85%, 89.14%, 93.13%, and 94.99% for TCE concentrations of 3000, 1500, 300 and 150 μg/L respectively. Accordingly, the degradation rate at this pressure was enhanced by approximately 30% compared to those conditions in which oxygen was not applied. As illustrated, the TCE removal rate did not change significantly above oxygen pressures of 2 atm. Isaev et al. have studied the influence of dissolved oxygen under pressure on electrochemical oxidation of toluene and acetone from aqueous solutions. They reported that the oxidation of toluene and acetone by electrolysis under oxygen pressure was accelerated due to the generation of active particles of the ions O_2_^-^ and HO_2_^-^, and radicals including HO_2_° and HO°. They also showed that increasing the oxygen pressure increased the efficiency of the overall process [20].

The increasing TCE degradation with increasing oxygen pressure in our present work can be attributed to the generation of OH° radicals due to the reaction between H_2_O_2_ and pressurized O_2_, similar to the reaction which occurs between H_2_O_2_ and O_3_ for the production of hydroxyl radicals as follows [21]:

(3) $$H_{2}O_{2}+2O_{3}\;\to \;OH^{\circ }+OH^{\circ }+3O_{2}$$

Therefore, the efficiency of hydroxyl radical production plays an important role in the degradation of TCE and PCE.

## Conclusions

In this study degradation of TCE was investigate in a stainless steel reactor with various experimental conditions including different H_2_O_2_ concentrations, TiO_2_ nano-particle dosage, pH, initial TCE concentration and different oxygen pressure. The results of this study show that the optimum conditions for TCE degradation are an H_2_O_2_ concentration of 50 mg/L, a TiO_2_ concentration of 100 mg/L, an acidic pH and a reaction time of 90 min. Additionally, the results indicated that under pressure dissolved oxygen has a synergistic effect on the H_2_O_2_/TiO_2_ AOP and enhances the degradation of TCE. The application of 2 atm of dissolved oxygen under optimized conditions improved the effectiveness of TCE degradation by 30%. This synergistic effect provides a promising technology for the removal of TCE and can also be used to remove other organic contaminants from aqueous solutions.

## Competing interests

The authors declare that they have no competing interests.

## Authors’ contributions

The overall implementation of this study including design, experiments and data an analysis, and manuscript preparation were the results of efforts by corresponding author. All authors have made extensive contribution into the review and finalization of this manuscript. All authors read and approved the final manuscript.
